# Supplementary material for: Association between oral microbiome and seven types of cancers in East Asian population: a two-sample Mendelian randomization analysis
Source: Front Mol Biosci. 2023 Nov 21;10:1327893. doi: 10.3389/fmolb.2023.1327893 (PMC10702768; doi:10.3389/fmolb.2023.1327893)
Supplement: Supplementary file 1 [file DataSheet1.ZIP › Supplementary Materials/MR plots of saliva/saliva═╝/ovarian cancer/pheno.2642_to_ovarian cancer_forest.pdf]

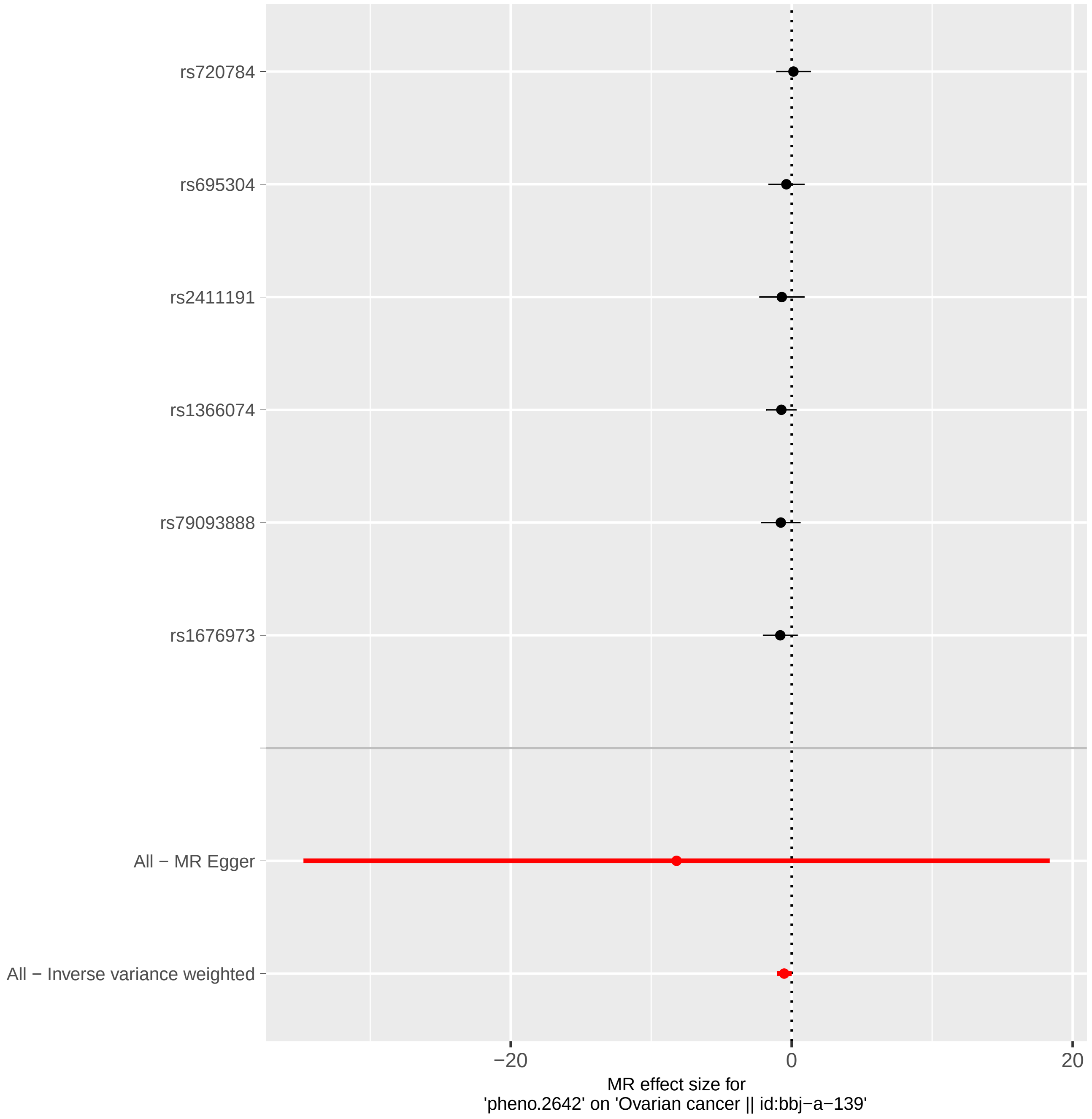

rs720784
rs695304
rs2411191
rs1366074
rs79093888
rs1676973
All − MR Egger
All − Inverse variance weighted
−20
0
20
MR effect size for
'pheno.2642' on 'Ovarian cancer || id:bbj−a−139'
